# Diversity and Function of Wolf Spider Gut Microbiota Revealed by Shotgun Metagenomics

**DOI:** 10.3389/fmicb.2021.758794

**Published:** 2021-12-17

**Authors:** Runbiao Wu, Luyu Wang, Jianping Xie, Zhisheng Zhang

**Affiliations:** ^1^Key Laboratory of Eco-environments in Three Gorges Reservoir Region (Ministry of Education), School of Life Sciences, Southwest University, Chongqing, China; ^2^Key Laboratory of Freshwater Fish Reproduction and Development (Ministry of Education), School of Life Sciences, Southwest University, Chongqing, China

**Keywords:** shotgun metagenomic sequencing, spiders, host-bacterial interaction, symbiosis, microbiome

## Abstract

Wolf spiders (Lycosidae) are crucial component of integrated pest management programs and the characteristics of their gut microbiota are known to play important roles in improving fitness and survival of the host. However, there are only few studies of the gut microbiota among closely related species of wolf spider. Whether wolf spiders gut microbiota vary with habitats remains unknown. Here, we used shotgun metagenomic sequencing to compare the gut microbiota of two wolf spider species, *Pardosa agraria* and *P. laura* from farmland and woodland ecosystems, respectively. The results show that the gut microbiota of *Pardosa* spiders is similar in richness and abundance. Approximately 27.3% of the gut microbiota of *P. agraria* comprises Proteobacteria, and approximately 34.4% of the gut microbiota of *P. laura* comprises Firmicutes. We assembled microbial genomes and found that the gut microbiota of *P. laura* are enriched in genes for carbohydrate metabolism. In contrast, those of *P. agraria* showed a higher proportion of genes encoding acetyltransferase, an enzyme involved in resistance to antibiotics. We reconstructed three high-quality and species-level microbial genomes: *Vulcaniibacterium thermophilum, Anoxybacillus flavithermus* and an unknown bacterium belonging to the family Simkaniaceae. Our results contribute to an understanding of the diversity and function of gut microbiota in closely related spiders.

## Introduction

Gut microbial communities can be transmitted vertically in spiders and their composition is influenced by the consumed prey (Kennedy et al., 2020; Sheffer et al., 2020). They may provide energy and contribute to defense against pathogens and parasites (Moran et al., 2019). With over 49,500 known species of spiders worldwide (World Spider Catalog, 2021), the diversity and function of the gut microbiota of spiders deserve greater attention. Spiders are characterized by their unique feeding strategies that are based on extraneous digestion and internal absorption, whereby they first bite into the body of their prey, inject digestive enzymes, and subsequently suck the digested contents. It has been demonstrated that the microbiota of spiders can play an important role in contributing to the spider’s survival, as it can affect digestion, metabolism, and the maintenance of health (Kumar et al., 2020). *Pardosa* spiders (Araneae: Lycosidae) are carnivorous and euryphagous arachnids that are generally ground-dwelling predators in agroecosystems and forests. They are well known for their application in pest control in farming. Therefore, it is of particular interest to analyze the composition, abundance, and function of the gut microbiome in *Pardosa* spiders.

Marker gene, metatranscriptome and whole metagenome analysis have been used to explore the microbial communities (Knight et al., 2018). The selection of sequencing method mainly depends on the scientific question and sample type (Liu et al., 2021). Marker genes including 16S ribosomal RNA (rRNA) amplified and sequenced for identifying bacteria and archaea, 18S rRNA and internal transcribed spacer (ITS) for identifying fungi sequencing. Using marker gene sequencing approach, species of *Wolbachia*, *Cardinium*, *Rickettsia*, *Spiroplasma*, *Rickettsiella* and some unidentified, possibly symbiotic bacteria have been detected in spider (Zhang et al., 2018b; Sheffer et al., 2020). The metatranscriptome sequencing technology is used to profile messenger RNA (mRNA) in microbiomes and the transcription of microbiome, which was limited by the complexity of sequencing and bioinformatic tools available. Whole metagenome analysis such as shotgun metagenomics is an untargeted sequencing method that all microbial genomes within a sample are sequenced simultaneously. Shotgun metagenomics has the potential not only to profile taxonomic composition and functional potential of microbial communities, but also to recover whole genome sequences (Quince et al., 2017). Given that the microbiota of several organisms has yet to be described, the metagenomic sequencing is appropriate for studying the gut microbiome of spiders.

The gut microbiome plays important roles in several spider traits, and recent studies have shown that spiders are hosts to many types of endosymbionts (Zhang et al., 2018b). Furthermore, it has been established that spiders on the same web are characterized by a very similar microbial richness, whereas differences in the microbial compositions are more pronounced among spiders on different webs (Busck et al., 2020). Kumar et al. (2020) found that the gut microbiota in the Oxyopidae and Thomisidae spider families not only metabolize fatty acids and sugars but are also involved in the synthesis of vitamins. Moreover, spiders have also been found to be reservoirs of genetically diverse viruses. For example, Debat (2017) identified the sequences of 10 viral species using the RNA data obtained from the spider *Nephila clavipes*.

In this study, we aimed to gain further insights into the composition and function of the gut microbiota of spiders. We adopted a metagenomic approach to establish whether gut microbial communities differ between two closely related *Pardosa* species in the *Pardosa laura* group, namely, *P. agraria and P. laura,* and further examined their functional roles. These two species are known for their different habitats, with *P. agraria* typically inhabiting cultivated areas, such as vegetable gardens, farmland, and artificial grassland, and *P. laura* prevalent in forests that are adjacent to farmlands. Moreover, the *Pardosa laura* group is one of the models for gut microbial studies because the samples are easy to collect. Adults of both species are found from May to September, and reproduce twice annually (Lu et al., 2021). The findings of this study will provide new insights into the diversity and function of the gut microbiota of spiders.

## Materials and Methods

### Sample Collection

**Figure 1:**
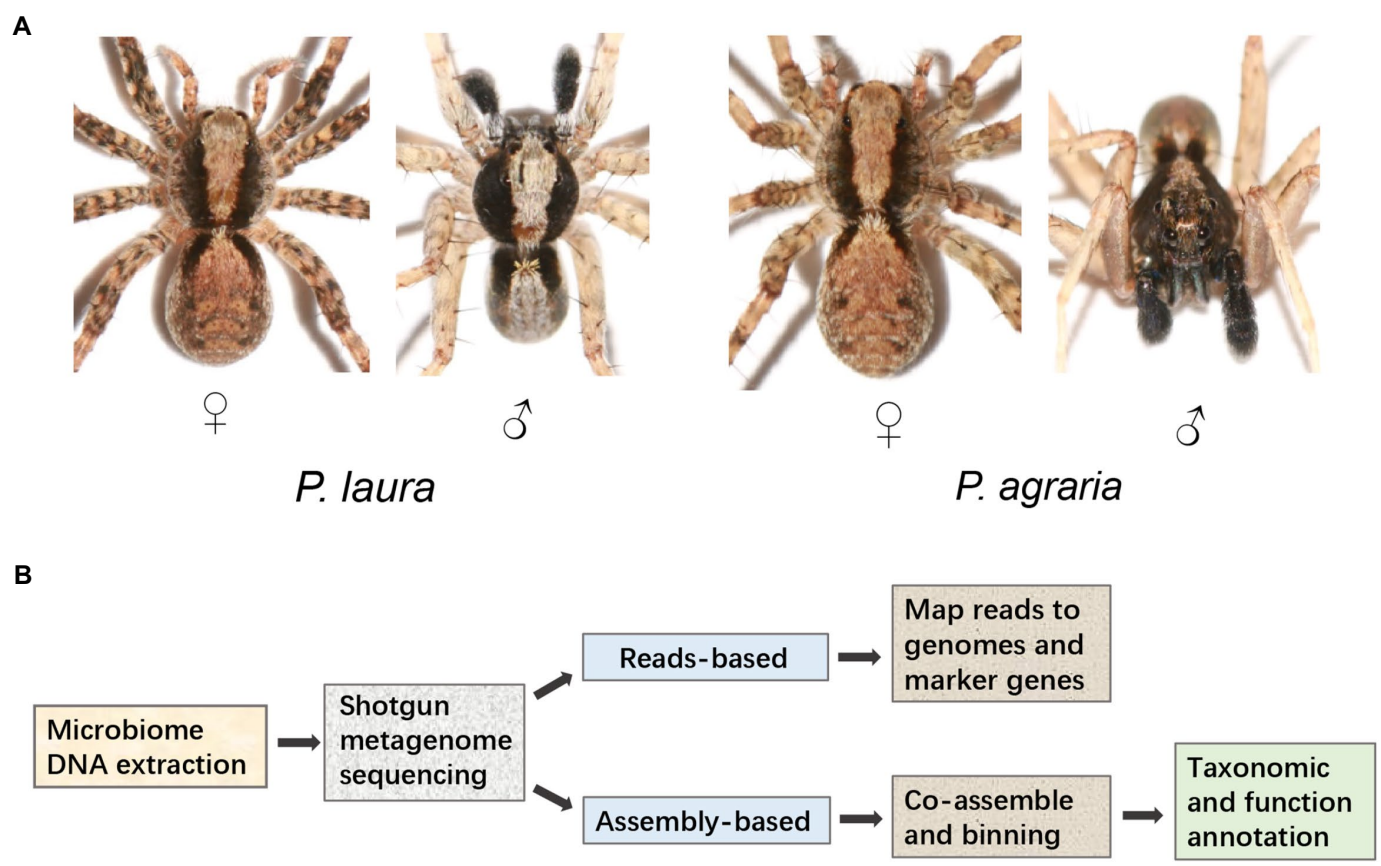
Study cohort and design. **(A)** Morphological characteristic of *Pardosa laura* and *Pardosa agraria* spiders. **(B)** Metagenomic analysis pipeline.

Specimens of *Pardosa laura* Karsch, 1879 and *Pardosa agraria* Tanaka, 1985 (*n* = 20 for each species) were collected for three consecutive years (June 5th, 2018; May 11th, 2019; and May 2nd, 2020) in the Jinyun Mountain Natural Reserve (29°50.5′N, 106°21.5′E; Alt. 248 m), from adjacent areas of woodland (*P. laura*) and farmland (*P. agraria*; Figure 1A). The collected specimens were placed in anhydrous ethanol and immediately stored at −20°C. The experimental design is shown in Figure 1B.

### DNA Extraction and Shotgun Sequencing

Following the manufacturer’s instructions, DNA was extracted from the gut contents of spiders using a DNeasy^®^ Blood and Tissue Kit (Qiagen, Germany). The spiders were dissected under a 205C microscope (Leica, Germany), and the gut contents were dissected from the opisthosoma using a dissecting needle. A total of six genomic DNA samples from the two species collected over the 3 years were extracted from the gut contents, and the total DNA obtained was measured using a Qubit 2.0 fluorometer (Life Technologies, United States).

High-quality genomic DNA was sheared into smaller fragments using the Covaris S/E210 ultrasonicator or Bioruptor. The sheared DNA was digested into blunt-ended fragments using T4 DNA polymerase, Klenow Fragment, and T4 polynucleotide kinase. Thereafter, an adenosine base was attached to the 3′ blunt end to facilitate the ligation of adapter sequences to the DNA fragments. Following PCR amplification and gel electrophoresis, fragments greater than 300 bp were retained for the construction of qualified libraries, which were subsequently sequenced at BGI (Wuhan, China) at 10 G and 150 PE using an Illumina Hiseq X ten sequencer (San Diego, United States).

### Assembly-Free Metagenomic Profiling

Bioinformatic analysis was carried out as per the procedure described by Liu et al. (2021). Quality control of metagenomic sequencing data was performed using FastQC v0.11.81 with parameter -t 20 and MultiQC v1.10.1 (Ewels et al., 2016) with parameter -d. Contaminating spider and human DNA were removed using the assembled whole-genome contig sequences of *Pardosa pseudoannulata* and the human genome (hg38) based on the KneadData pipeline,^1^ which integrates the KneadData v0.6.1, Trimmomatic v0.39, and Bowtie2 v2.3.5 software programs with parameters – threads 20 – serial – run-trf – remove-intermediate-output – bowtie2-options “– very-sensitive – dovetail.” Microbial abundance was calculated using the kraken2 v2.1.2 software (Wood et al., 2019) using a pre-built kraken 2 database (minikraken_8GB_202003)^2^ with parameters – threads 40 – use-names – report-zero-counts. This program is based on the k-mer algorithm that can be used to rapidly classify sequenced reads to the species level. Thereafter, we utilized Bracken v2.5.0 (Lu et al., 2017) with parameters -r 150 -t 0 to enhance the accuracy of estimate of the abundance of each taxa. Finally, the phylum-level comparisons were plotted using *ggplot2* package (Villanueva and Chen, 2019) in statistical computing environment (R v4.1.0; R Core Team, 2013).

### Metagenome Assembly, Gene Prediction, and Annotation

*De Novo* co-assembly of the genome was carried out using MEGAHIT v1.2.9 (Li et al., 2016) with parameters – k-min 27 – k-max 127 – k-step 10. Compared with the metaSPAdes software, MEGAHIT requires less computing memory and assembly can be carried out more rapidly. The genes associated with contigs were predicted using prodigal v2.6.3 (Hyatt et al., 2010) with default parameters, and duplicates were removed using CD-HIT v4.8.1(Fu et al., 2012) with parameters -aS 0.9 -c 0.95 -G 0 -g 0. Gene quantification was performed using salmon v1.4.0 (Patro et al., 2017; run with the default parameters). Moreover, functional genes were annotated based on eggNOG database^3^ using eggNOG-mapper v2.0.1 (Huerta-Cepas et al., 2019) with parameters – no_annot – no_file_comments – override – data_dir eggnog5 – cpu 60 -m diamond. The Gene Ontology (GO) analysis was conducted by R package *clusterProfiler* v4.0 (Wu et al., 2021). The numbers of Kyoto Encyclopedia of Genes and Genomes (KEGG) Orthologs (KO) for each pathway were performed *via* the website https://www.kegg.jp/ghostkoala/ (Kanehisa et al., 2016).

### Analysis of Genes Related to Carbohydrate and Antibiotic Resistance

Carbohydrate active enzymes (CAZymes) are essential microbial enzymes that play roles in hydrocarbon metabolism. We annotated CAZymes for the newly sequenced genomes based on diamond searches of the dbCAN2 database (Zhang et al., 2018a) with parameters – outfmt 6 – sensitive -e 1e-5 – max-target-seqs 1 – quiet. The number of extracted carbohydrate genes was calculated using the python script, and differences between groups were visualized by comparing gene abundance using the STAMP software (Parks et al., 2014).

As antibiotic resistance becomes more prevalent, the environmental factors contributing to the spread of antibiotic resistance genes are intensively investigated beyond the clinical realm (Li et al., 2020). The antibiotic resistance genes of spider gut microbiota were assessed based on a diamond search against the Resfams profile HMM database (Gibson et al., 2015).

### Contig Binning

Initially, contigs shorter than 1,000 nt were discarded based on screening using seqtk v1.3-r106 (Li, 2012) with the parameter seq -L 1,000. The assembled contigs were simultaneously binned using CONCOCT (Alneberg et al., 2014), MaxBin 2 (Wu et al., 2016), and MetaBAT2 (Kang et al., 2019) in metaWRAP v1.3.2 (Uritskiy et al., 2018) with the parameter -t 60, which provides an easy-to-use metagenomic analysis suite. Having binned the contigs, the genomes of the strains were obtained by clustering contigs of similar composition or abundance.

### Quality Control, Classification, and Annotation

Putative genomes were subjected to quality control to generate the final draft genomes. Those genomes with >50% completeness and < 10% contamination were selected using the bin_refinement module in metaWRAP with parameters -c 50 × 10 -t 60. All genomes were de-replicated using dRep v3.2.0 (Olm et al., 2017) with the parameters -sa 0.95 -nc 0.30 -comp 50 -con 10 -p 3, after which additional re-assembly was performed to enhance the quality of assembled bins using the re-assemble_bins module with parameters -t 96 -m 800 -c 50 × 10. We also ran the classify_bins module and annotate_bins module to determine the taxonomy and to functionally annotate the re-assembled bins. Furthermore, to classify bins, we used the toolkit GTDBTk v1.5.1 (Chaumeil et al., 2020) with parameters – extension fa – prefix tax – cpus 24, which is dependent on the genome taxonomy database (GTDB) containing 258,406 genomes of bacteria and archaea (Parks et al., 2020).

### Phylogenetic Analysis

To reconstruct the phylogenetic relationships, a total of 31 genomic sequences (two *Vulcaniibacterium thermophilum*, twenty-eight *Anoxybacillus flavithermus*, one *Candidatus Neptunochlamydia vexilliferae*) were downloaded from the NCBI database (Supplementary Table1). We constructed a phylogenetic tree based on 120 single-copy genes of 34 genomic sequences. The sequences alignment and phylogenetic tree construction were performed by the software GTDBTk v1.5.1 (Chaumeil et al., 2020) with parameters infer – prefix tax – cpus 24. Finally, phylogenetic trees were visualized and annotated in iTOL v 6.4 (Letunic and Bork, 2021).

### Statistical Analysis

The alpha diversity and beta diversity analysis were conducted using the R programming language. The species-level bacterial richness measures (richness, chao and ACE) and bacterial diversity measures (shannon, simpson and invsimpson) based on ANOVA/Tukey’s HSD test statistical methods in the spider groups were calculated with R package *amplicon* (Zhang et al., 2019; Liu et al., 2021). The differences between the two spider groups were considered significant when *p* < 0.05 and the comparative results were visualized using boxplots. The beta diversity metrics was calculated using USEARCH (Alloui et al., 2015) with the parameter -beta_div. Principal coordinates analysis (PCoA) based on the Bray-Curtis distance matric was performed to explore the differences in bacterial communities at the species level between samples. Adonis (PERMANOVA) testing was utilized to confirm significant differences in microbial community. We performed metaMDS analysis to implement non-metric multidimensional scaling (nmds) based on the Bray-Curtis distance matric using the *vegan* package in R (Oksanen et al., 2007).

## Results

### Identification of Microbiota From the Reads of Spider Metagenomes

**Figure 2:**
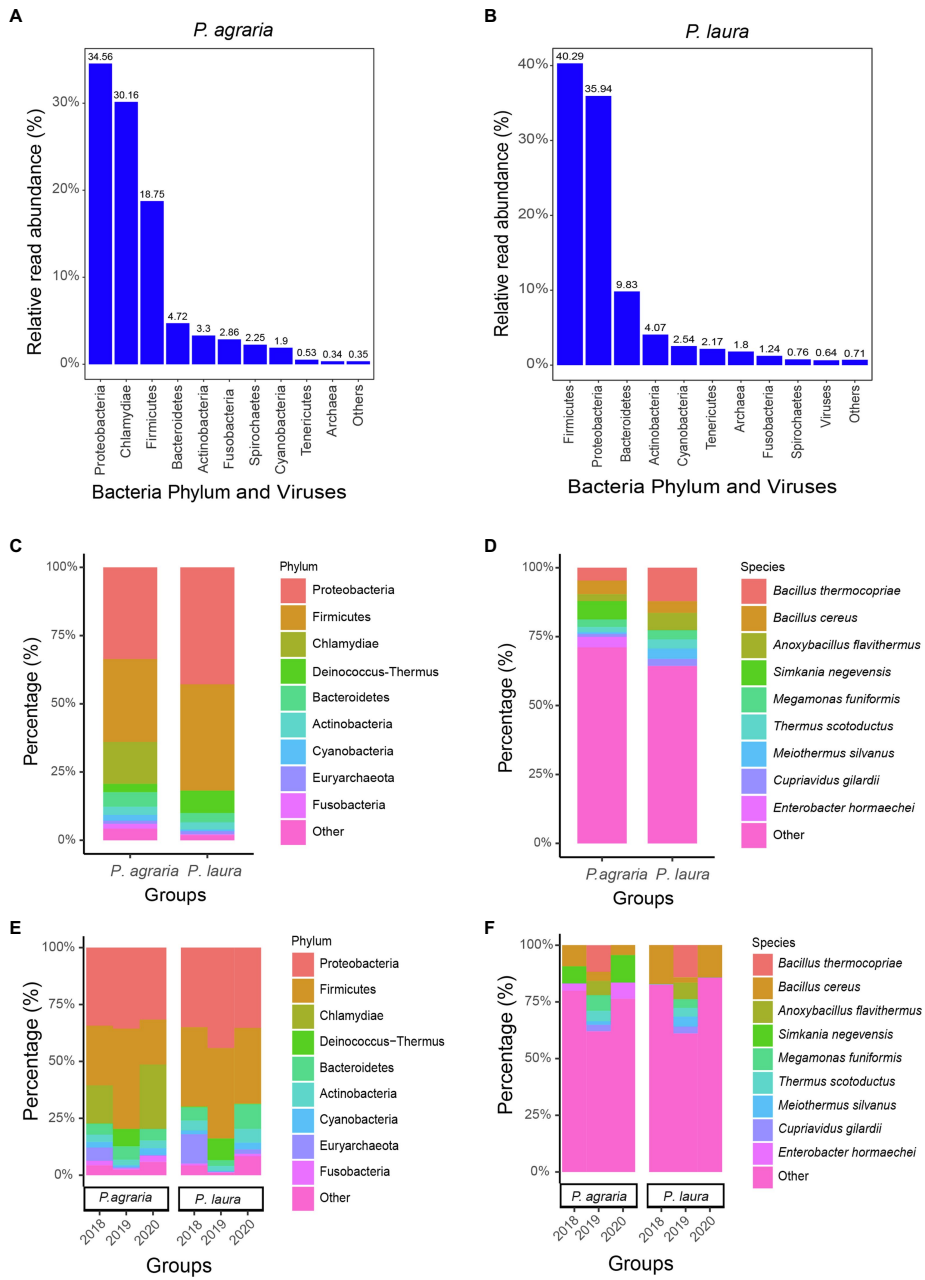
Gut microbiome composition of the two *Pardosa* species. The relative read abundance comparisons by bacteria phylum and viruses for *Pardosa agraria* **(A)** and *Pardosa laura* **(B)**. Relative percentages (for the period 2018–2020) for microbiome at the phylum **(C)** and species **(D)** levels based on mixed sequencing of the gut contents of 20 spiders, and their relative percentages for each year at the phylum **(E)** and species **(F)** levels.

Following quality control and the removal of sequences of human and spider origin, we obtained 35,813,674 to 51,483,599 (average 41,003,015) high-quality reads. The most abundant gut microbiota in *P. agraria* were bacteria, accounting for 99.64%, followed by archaea and viruses accounting for 0.34 and 0.02%, respectively (Figure 2A). In the gut of P. laura, bacteria, archaea, and viruses accounted for 97.56, 1.8, and 0.64%, respectively (Figure 2B). In both species, bacteria from the phylum Proteobacteria were most abundant, accounting for approximately 27.3% (127,304/466,552) in *P. agraria* and 34.4% (186,388/542,484) in *P. laura* (Figure 2C). Firmicutes, the second most abundant group in both *P. agraria* and *P. laura*, accounted for at 24.6% (114,998/466,552) and 31.1% (168,926/542,484), respectively. However, bacteria in the phylum *Chlamydiae* were detected only in *P. agraria*. We found that more than 60% of the sequenced microbial reads could not be mapped at the species level (Figure 2D).

To investigate whether the gut microbiota of spiders is consistent, we compared the gut microbial composition of spiders collected between 2018 and 2020. The results showed that the gut microbial composition of spiders within the same habitat was similar at the phylum level (Figure 2E). However, at the species level, the relative abundance of microorganisms showed some fluctuations in spiders collected in 2019 (Figure 2F). The number of reads of *Bacillus cereus* was the highest among the gut microbiota in both spider species.

**Figure 3:**
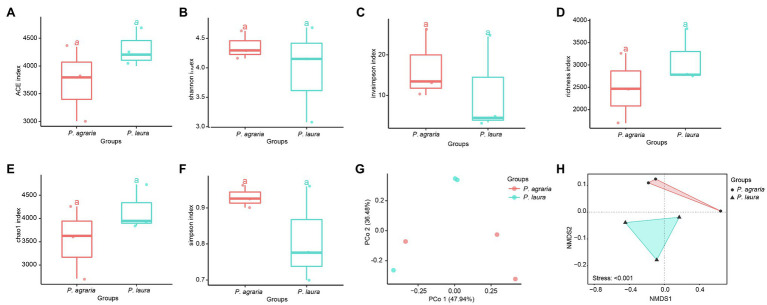
Alpha-diversity at the species level of the gut microbial community between the two *Pardosa* species based on **(A)** ACE, **(B)** shannon, **(C)** invsimpson, **(D)** richness, **(E)** chao1 and **(F)** simpson metrices. Each point represents a sample, and three samples of each spider species were collected in 2018, 2019 and 2020. Significance between the spider groups is based on ANOVA/Tukey’s HSD test statistical methods. PCoA (Bray-Curtis metric) for visualization of gut microbial community dissimilarities. Samples colored by spider group **(G)** (*R*^2^ = 0.047; *p* = 0.9). The distance between two points represents their level of similarity. NMDS of occurrence-based Bray–Curtis dissimilarity of microbial species level communities from two *Pardosa* species **(H)**.

The alpha diversity analysis revealed no significant differences between the alpha diversity analysis between the gut microbiota of *P. agraria* and *P. laura* (Figures 3A–F). These results suggest that the two species share a similar gut microbial abundance. In contrast, we detected certain differences in the species richness, as indicated by the beta diversity analysis (Figures 3G,H).

### Gene Occurrence and Functional Annotation of Assembled Metagenomes

**Figure 4:**
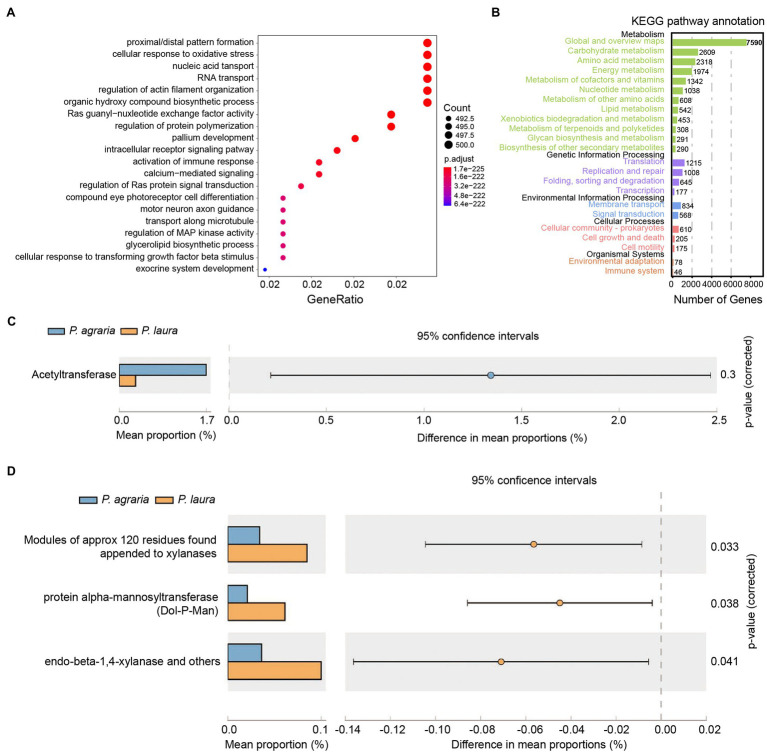
Functional annotation of the genes in gut bacteria of the two *Pardosa* species. **(A)** Genes that mapped to the COG category were annotated using eggnog. Top 20 of the categorized functional annotation enrichment predicted in the bubble chart. The size of the circle indicates the number of genes and the color represents the significance. **(B)** Kyoto Encyclopedia of Genes and Genomes functional category analysis was done using GhostKOALA. Rows indicate the number of genes annotated to the corresponding B-level pathway, columns in black font are the name of the classification. **(C)** Comparison of antibiotic resistance-associated genes in the two *Pardosa* species based on the resfam database. **(D)** Comparison of carbohydrate metabolism-associated genes in the two *Pardosa* species based on the carbohydrate-active enzyme database(dbCAN2).

Assembly of the metagenomic reads from all samples yielded a length of 1,398,928,808 nt, in which 767,579 contigs, and 443,352 unique genes were detected based on annotation. The results of COG enrichment analysis revealed that genes from the microbiota of the two *Pardosa* spiders have similar functional activities, i.e., synthesis, regulation, oxygen response, and immune response (Figure 4A). 12,098 genes were annotated and mapped to the KEGG pathway, and the number of genes annotated to the carbohydrate, amino acid, and energy metabolism pathways ranked to be the top three (Figure 4B). The analysis of antibiotic resistance indicated that the gut microbiota of *P. agraria* was characterized by a significantly higher abundance of acetyltransferases compared with that of *P. laura* spiders (Figure 4C). On comparing carbohydrate-metabolism genes in the gut microbiota based on dbCAN2 database, the modules of carbohydrates were found to be significantly higher in *P. laura* than in *P. agraria*. Such modules of approximately 120 residues were discovered attached to enzymes such as xylanases, protein alpha-mannosyltransferase, and endo-beta-1,4-xylanase (Figure 4D).

### Functional Analysis of Assembled Bins and Phylogenetic Relationships

**Figure 5:**
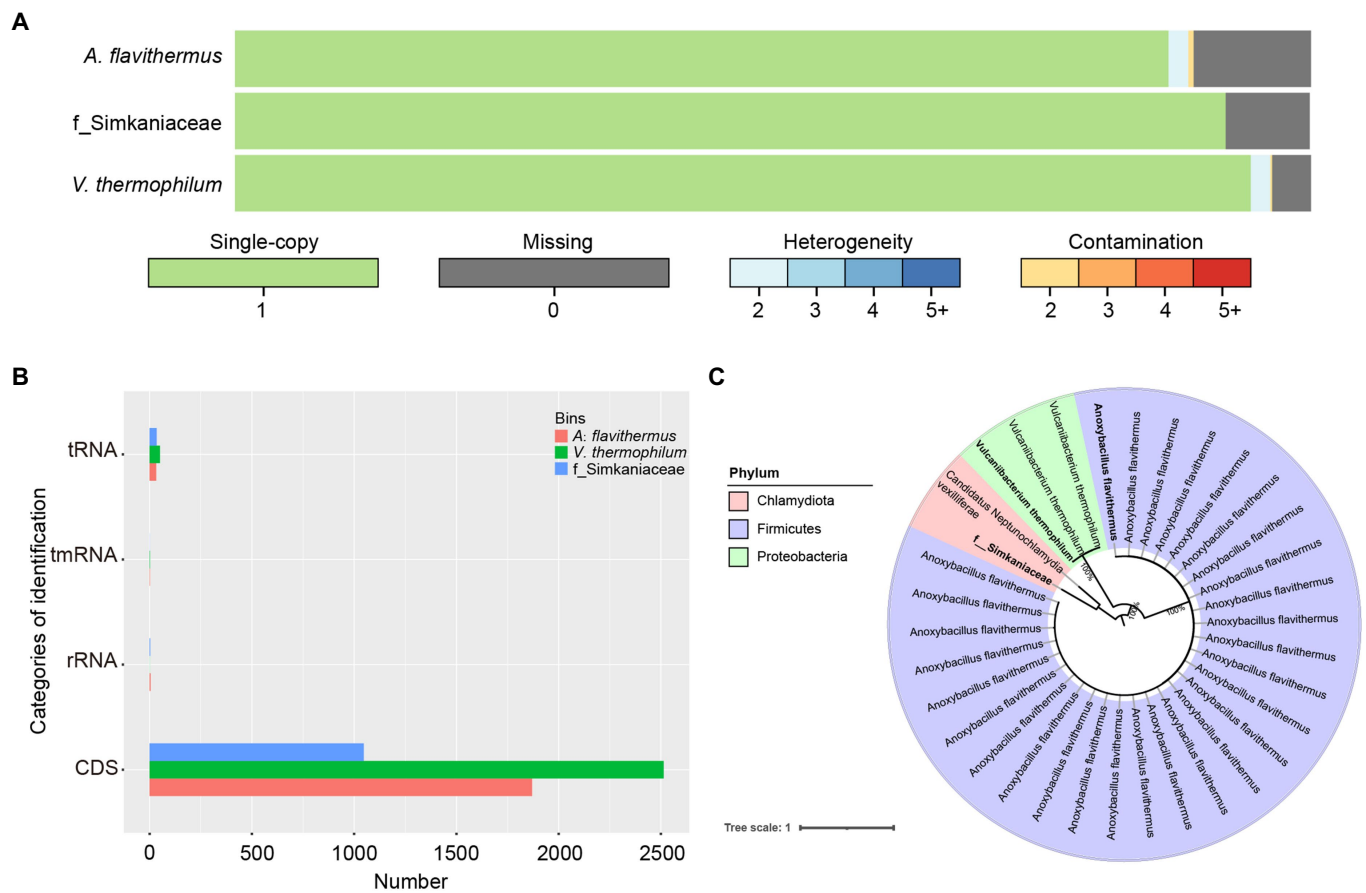
Functional analysis of the individual bacterial bins. **(A)** Visualization of three bins obtained after quality control by CheckM and de-duplication by Drep, with single-copy genes in green, deletion genes in gray; blue gradient represents various heterozygosity rates; red represents contamination rates. **(B)** Comparison of the number of identifications among tRNA, tmRNA, rRNA and CDS. **(C)** Phylogenetic relationships of 29 *Anoxybacillus flavithermus*, three *Vulcaniibacterium thermophilum*, and two bacteria belonging to family Simkaniaceae were investigated using maximum-likelihood method. Three bacterial names in bolded were assembled in this study. Numbers at each node showed ML bootstrap value.

Having binned contigs using CONCOCT, MaxBin2, and MetaBAT2, we successfully reconstructed three high-quality species-level genomic bins (SGBs). Among these, the genome of *Vulcaniibacterium thermophilum* reached 95.43% completeness, with a length of 2,675,526 nt, whereas *Anoxybacillus flavithermus* with a length of 1,795,422 nt was 83.11% complete. The third SGB was that of an unidentified bacterium within the family Simkaniaceae, which was 90.2% complete with a length of 1,158,258 nt (Figure 5A). Functional annotation of the SGBs using prokka in the annotate_bins module, revealed that the *V. thermophilum* genome contains 2,513 coding sequences (CDS), one tmRNA, and 50 tRNAs and indicated that this bacterium can degrade chlorinated phenols and other aromatic compounds (Figure 5B). *A. flavithermus* contains 1,870 CDS, one tmRNA, 32 tRNAs, and four rRNAs, whereas the Simkaniaceae species contains 1,047 CDS, 34 tRNAs, and three rRNAs. Thus, these genes participate in diverse biological functions. To further examine the relationship among the assembled genomes of gut microbiota, we reconstructed a phylogenetic tree based on 120 single-copy genes from 34 assembled genomes. As shown in Figure 5C, three SGBs belong to different phyla and one of which was not mapped to known species. Additionally, the phylogenetic topologies also showed that *V. thermophilum* and *A. flavithermus* had high maximum-likehood (ML) bootstrap values (=1) with the same species, respectively. The third SGB was closely related to *Candidatus Neptunochlamydia vexilliferae*, and they both belong to the family Simkaniaceae.

## Discussion

In this study, we sequenced the gut microbiota of the congeneric spiders *P. agraria* and *P. laura* using a metagenomic approach to evaluate the extent of microbial diversity. Given that metagenomic sequencing can provide an accurate indication of the true composition of the sampled microbiota, we established that spiders in the genus *Pardosa* have a rich gut microbial population. Moreover, although we detected similar abundances in the microbiota across the two species, the gut microbiota of the forest-inhabiting *P. laura* was found to show a greater species richness than that of *P. agraria,* which was collected from adjacent farmlands. Gene functional annotation indicated that the gut microbiota of *P. laura* has a greater ability to utilize polysaccharides for energy supplementation. Our findings highlight the utility of metagenomic sequencing technology in studying the gut microbiota of spiders.

The reference database is important for the classification of microbial species, but there is no complete and exclusive reference database for spider gut microbiome. In this study, we download the standard Kraken 2 database used for the taxonomic classification because this database is built from the complete genomes in RefSeq for the bacteria, archaea and viral libraries, as well as NCBI taxonomic information. Some microbiomes are not yet included in the reference database cannot be identified in our study (false negatives) since the reference database affects the accuracy of microbial classification. As microbial reference databases continue to improve, the accuracy of mapping-based annotation methods will become increasingly accurate, and metagenomic technologies have great potential for future applications. In practical studies, we recommend choosing the same reference database when we want to compare gut microbiota of different species.

Hu et al. (2019) identified a total of 150 families and 23 phyla of microbiota based on 16S rRNA sequencing from guts of the three spider species *Pardosa laura*, *Pardosa astrigera*, and *Nurscia albofasciata*. In this study, we successfully identified more than 47 bacterial phyla within the gut microbiota of two *Pardosa* species using metagenomic analysis. These taxonomic assignments were based on the currently available reference datasets; thus, it is highly probable that the true diversity of the gut microbiome of the two species exceeds that determined in the present study. We were also able to sensitively identify the top five most abundant microbiota in the *Pardosa* gut at the phylum level, namely Proteobacteria, Actinobacteria, Firmicutes, Chlamydiae, and Bacteroidetes. Kumar et al. (2020) detected a lower microbial diversity (16 phyla) in the gut microbiota of spiders in the Thomisidae and Oxyopidae families. Consistent with the findings of the present study, Proteobacteria and Firmicutes have previously been reported as the most abundant gut microbial phyla in more than 10 species of spiders.

We are very interested in the biological and ecological implications of the top 9 species found. The top 9 species are *Bacillus thermocopriae, Bacillus cereus, Anoxybacillus flavithermus, Simkania negevensis, Megamonas funiformis, Thermus scotoductus, Meiothermus silvanus, Cupriavidus gilardii, Enterobacter hormaechei.* Bacteria are important ecological indicators. *Bacillus thermocopriae*, Gram-positive, facultatively anaerobic, motile, endospore-forming, rod-shaped strain, was firstly proposed as a new species in 2013 when isolated from a windrow compost pile and characterized by means of a polyphasic approach (Han et al., 2013). A strain of *Bacillus thermocopriae,* namely moderate thermotolerant *Bacillus thermocopriae* IR-1, was isolated from impact crater soil immediately after the meteorite impact event Mukundpura, India, on June 6, 2017 (Thombre et al., 2019). *Bacillus thermocopriae* IR-1 was a member of the microbial community that survived the meteorite impact. The presence of a species that survived the effect of shock waves at a peak shock pressure of 300 kPa, temperature 400 K, and Mach number of 1.47 in the spider is very interesting. This species was also found on Manasbal Lake, the deepest spring fed valley lake of Kashmir (Shafi et al., 2017). There are only 3 papers documented very few information on this species, most are related to heat niche. The first-time characterization of this species from spider implicates a unique ecological role of this species in spider habitat adaptation. Further studies on the heat-resistant enzymes of this species are worthwhile. *Bacillus cereus* is an opportunistic pathogen causing food intoxication and infectious diseases. There are many studies on its biology, epidemiology, and pathogenesis (Enosi Tuipulotu et al., 2021), but nearly no information on spider *B.cereus*. The high abundance of this species might confer some ecological advantage to the host. *Anoxybacillus flavithermus* species is spore-forming bacteria found ubiquitously in natural thermophilic environments, and can produce compounds and enzymes with ecological and industrial values. Spores produced by *A. flavithermus* can survive harsh environmental conditions encountered. Further elucidation its metabolism for spider ecological adaptation will be very instructive (Khalil et al., 2019). *Simkania negevensis* is emerging Chlamydia-related obligate intracellular bacteria discovered in 1993 and represents the founding member of the Simkaniaceae family within the Chlamydiales order. Like the pathogenic organisms *Chlamydia pneumoniae* and *Chlamydia trachomatis*, *Simkania negevensis* has biphasic developmental cycle causes acute and chronic human genital infections and respiratory diseases. Its hosts range from unicellular amoeba to a variety of human cells, such as epithelial HeLa and macrophage-like THP1 cells. *S. negevensis* grows in amoeba or human cells within a membrane-bound vacuole (Simkania-containing vacuole, SnCV) forming endoplasmic reticulum (ER) contact sites. The SnCV membrane is an interface critical for host-pathogen interaction. *S. negevensis* exploits early endosome-to-trans-Golgi network interface (retrograde transport) for nutrient acquisition and growth, and to affect the SnCV formation, morphology and lipid transport (Herweg et al., 2016). *S. negevensis* has a life cycle more than 12 days (Koch et al., 2020), which will be very interesting to explore whether this holds true in spiders. *Megamonas funiformis* was firstly proposed as a new species in 2008, when isolated from human faeces as anaerobic, non-spore-forming, Gram-negative (Sakon et al., 2008). 2011, it was found in pig faeces (Jeong et al., 2011). We presume that human or animal excrement is present in the spider’s habitat. *Thermus scotoductus* preferentially colonizes water heaters at the expense of local environmental Thermus strains (Wilpiszeski et al., 2019). Further isolation *Thermus scotoductus* phage (Plotka et al., 2015) from spider can promote our understanding of this bacteria. *Meiothermus silvanus* is extremely thermophilic Thermus with global genome rearrangements for the evolution at both genomic and metabolic network levels (Kumwenda et al., 2014). *Cupriavidus gilardii* is an emerging multidrug-resistant Gram-negative pathogen found in many environments (Ruiz et al., 2019). It is Cu-resistant (Huang et al., 2019). The presence of highly abundant *C. gilardii* in spider might indicate the antibiotics and copper pollution in the niche. Extended-spectrum beta-lactamase-producing Enterobacteriaceae identified in diverse ecosystem constitutes a serious ecological issue (Goldberg et al., 2019), as is true for the finding of *Enterobacter hormaechei* in spider. In summary, the most abundant species found in spider indicate a polluted and thermophilic niche. Further biochemical characterization of the spider can corroborate the microbiome discovery.

We also identified certain functional differences in the gut microbiota of the two *Pardosa* species, with microbes in the gut of *P. laura* tending to show more pronounced carbohydrate metabolism. Recent research has revealed that at least a proportion of species within the gut microbiota of spiders have evolved symbiotically and have been acquired partly through the ingestion of prey and partly by parental transmission or from the environment (Zhang et al., 2018b; Kennedy et al., 2020; Kumar et al., 2020; Sheffer et al., 2020). Our results demonstrate that *P. laura* preys on a wider range of species than *P. agraria*, and experiences greater competition for available food sources in the field. Consequently, a greater diversity of gut microbes may contribute to more efficient digestion and utilization of food. We also discovered that members of the *P. agraria* gut microbiota may play functional roles in antibiotic resistance and believe that this may be linked to the fact that *P. agraria* inhabits agricultural fields in which chemicals, such as pesticides and antibiotics, are likely to be used. Consequently, we consider that the wide variety of microbiota in the guts of wolf spiders contribute to digestion, energy metabolism, and even resistance to pathogens (i.e., nematodes, viruses, and fungi). We assembled only three high-quality microbial genomes from the 10G metagenomic sequencing data, and future studies could increase the sequencing depth to obtain more microbial genomes from the spider gut.

## Data Availability Statement

The datasets presented in this study can be found in online repositories. The names of the repository/repositories and accession number(s) can be found at: PRJNA750644, SRX11620190-SRX11620195.

## Author Contributions

RW performed the experiments, bioinformatic analysis, and wrote the original draft. LW collected and morphologically identified the specimen. JX revised the manuscript. ZZ is responsible for project management and funding application. All authors have read and agreed to publish the manuscript.

## Funding

This study was supported by the Key Natural Science Foundation of Chongqing (grant number cstc2019jcyj-zdxmX0006), the National Natural Science Foundation of China (grant numbers 31672278, 31702005), the Fundamental Research Funds for the Central Universities (grant number SWU120051).

## Conflict of Interest

The authors declare that the research was conducted in the absence of any commercial or financial relationships that could be construed as a potential conflict of interest.

## Publisher’s Note

All claims expressed in this article are solely those of the authors and do not necessarily represent those of their affiliated organizations, or those of the publisher, the editors and the reviewers. Any product that may be evaluated in this article, or claim that may be made by its manufacturer, is not guaranteed or endorsed by the publisher.

## Footnotes

^1^ http://www.bioinformatics.babraham.ac.uk/projects/fastqc/

^2^ ftp://ftp.ccb.jhu.edu/pub/data/kraken2_dbs/

^3^ http://eggnog5.embl.de

## References

[ref1] Alloui T. Boussebough I. Chaoui A. Nouar A. Z. Chettah M. C. (2015). “Usearch: A meta search engine based on a new result merging strategy”, in *2015 7th International Joint Conference on Knowledge Discovery, Knowledge Engineering and Knowledge Management (IC3K): IEEE*; November 12, 2015, 531–536.

[ref2] Alneberg J. Bjarnason B. S. de Bruijn I. Schirmer M. Quick J. Ijaz U. Z.. (2014). Binning metagenomic contigs by coverage and composition. Nat. Methods 11, 1144–1146. doi: 10.1038/nmeth.3103, PMID: 25218180

[ref3] Busck M. M. Settepani V. Bechsgaard J. Lund M. B. Bilde T. Schramm A. (2020). Microbiomes and specific symbionts of social spiders: compositional patterns in host species, populations, and nests. Front. Microbiol. 11:1845. doi: 10.3389/fmicb.2020.01845, PMID: 32849442 PMC7412444

[ref4] Chaumeil P. A. Mussig A. J. Hugenholtz P. Parks D. H. (2020). GTDB-Tk: a toolkit to classify genomes with the genome taxonomy database. Bioinformatics 36, 1925–1927. doi: 10.1093/bioinformatics/btz848 PMC7703759 31730192

[ref5] Debat H. J. (2017). An RNA Virome associated to the Golden orb-weaver spider Nephila clavipes. Front. Microbiol. 8:2097. doi: 10.3389/fmicb.2017.02097, PMID: 29118750 PMC5660997

[ref6] Enosi Tuipulotu D. Mathur A. Ngo C. Man S. M. (2021). Bacillus cereus: epidemiology, virulence factors, and host-pathogen interactions. Trends Microbiol. 29, 458–471. doi: 10.1016/j.tim.2020.09.003, PMID: 33004259

[ref7] Ewels P. Magnusson M. Lundin S. Kaller M. (2016). MultiQC: summarize analysis results for multiple tools and samples in a single report. Bioinformatics 32, 3047–3048. doi: 10.1093/bioinformatics/btw354, PMID: 27312411 PMC5039924

[ref8] Fu L. Niu B. Zhu Z. Wu S. Li W. (2012). CD-HIT: accelerated for clustering the next-generation sequencing data. Bioinformatics 28, 3150–3152. doi: 10.1093/bioinformatics/bts565, PMID: 23060610 PMC3516142

[ref9] Gibson M. K. Forsberg K. J. Dantas G. (2015). Improved annotation of antibiotic resistance determinants reveals microbial resistomes cluster by ecology. ISME J. 9, 207–216. doi: 10.1038/ismej.2014.106, PMID: 25003965 PMC4274418

[ref10] Goldberg D. W. Fernandes M. R. Sellera F. P. Costa D. G. C. Loureiro Bracarense A. P. Lincopan N. (2019). Genetic background of CTX-M-15-producing Enterobacter hormaechei ST114 and Citrobacter freundii ST265 co-infecting a free-living green turtle (*Chelonia mydas*). Zoonoses Public Health 66, 540–545. doi: 10.1111/zph.12572, PMID: 30843359

[ref11] Han L. Yang G. Zhou X. Yang D. Hu P. Lu Q.. (2013). Bacillus thermocopriae sp. nov., isolated from a compost. Int. J. Syst. Evol. Microbiol. 63, 3024–3029. doi: 10.1099/ijs.0.046953-0, PMID: 23396718

[ref12] Herweg J. A. Pons V. Becher D. Hecker M. Krohne G. Barbier J.. (2016). Proteomic analysis of the Simkania-containing vacuole: the central role of retrograde transport. Mol. Microbiol. 99, 151–171. doi: 10.1111/mmi.13222, PMID: 26374382

[ref13] Hu G. Zhang L. Yun Y. Peng Y. (2019). Taking insight into the gut microbiota of three spider species: No characteristic symbiont was found corresponding to the special feeding style of spiders. Ecol. Evol. 9, 8146–8156. doi: 10.1002/ece3.5382, PMID: 31380078 PMC6662400

[ref14] Huang N. Mao J. Zhao Y. Hu M. Wang X. (2019). Multiple transcriptional mechanisms collectively mediate copper resistance in Cupriavidus gilardii CR3. Environ. Sci. Technol. 53, 4609–4618. doi: 10.1021/acs.est.8b06787, PMID: 30920814

[ref15] Huerta-Cepas J. Szklarczyk D. Heller D. Hernandez-Plaza A. Forslund S. K. Cook H.. (2019). eggNOG 5.0: a hierarchical, functionally and phylogenetically annotated orthology resource based on 5090 organisms and 2502 viruses. Nucleic Acids Res. 47, D309–D314. doi: 10.1093/nar/gky1085, PMID: 30418610 PMC6324079

[ref16] Hyatt D. Chen G. L. LoCascio P. F. Land M. L. Larimer F. W. Hauser L. J. (2010). Prodigal: prokaryotic gene recognition and translation initiation site identification. BMC Bioinfo. 11, 1–11. doi: 10.1186/1471-2105-11-119, PMID: 20211023 PMC2848648

[ref17] Jeong J. Y. Park H. D. Lee K. H. Weon H. Y. Ka J. O. (2011). Microbial community analysis and identification of alternative host-specific fecal indicators in fecal and river water samples using pyrosequencing. J. Microbiol. 49, 585–594. doi: 10.1007/s12275-011-0530-6, PMID: 21887641

[ref18] Kanehisa M. Sato Y. Morishima K. (2016). BlastKOALA and GhostKOALA: KEGG tools for functional characterization of genome and metagenome sequences. J. Mol. Biol. 428, 726–731. doi: 10.1016/j.jmb.2015.11.006, PMID: 26585406

[ref19] Kang D. D. Li F. Kirton E. Thomas A. Egan R. An H.. (2019). MetaBAT 2: an adaptive binning algorithm for robust and efficient genome reconstruction from metagenome assemblies. Peerj 7:e7359. doi: 10.7717/peerj.7359, PMID: 31388474 PMC6662567

[ref20] Kennedy S. R. Tsau S. Gillespie R. Krehenwinkel H. (2020). Are you what you eat? A highly transient and prey-influenced gut microbiome in the grey house spider Badumna longinqua. Mol. Ecol. 29, 1001–1015. doi: 10.1111/mec.15370, PMID: 32011756

[ref21] Khalil A. B. Qarawi S. Sivakumar N. (2019). Genomic comparison of Anoxybacillus flavithermus AK1, a thermophilic bacteria, with other strains. Enzym. Microb. Technol. 131:109385. doi: 10.1016/j.enzmictec.2019.109385, PMID: 31615674

[ref22] Knight R. Vrbanac A. Taylor B. C. Aksenov A. Callewaert C. Debelius J.. (2018). Best practices for analysing microbiomes. Nat. Rev. Microbiol. 16, 410–422. doi: 10.1038/s41579-018-0029-9, PMID: 29795328

[ref23] Koch R. D. Horner E. M. Munch N. Maier E. Kozjak-Pavlovic V. (2020). Modulation of host cell death and lysis are required for the release of *Simkania negevensis*. Front. Cell. Infect. Microbiol. 10:594932. doi: 10.3389/fcimb.2020.594932, PMID: 33194844 PMC7658264

[ref24] Kumar V. Tyagi I. Tyagi K. Chandra K. (2020). Diversity and structure of bacterial communities in the gut of spider: Thomisidae and Oxyopidae. Front. Ecol. Evol. 8:351. doi: 10.3389/fevo.2020.588102

[ref25] Kumwenda B. Litthauer D. Reva O. (2014). Analysis of genomic rearrangements, horizontal gene transfer and role of plasmids in the evolution of industrial important Thermus species. BMC Genomics 15:813. doi: 10.1186/1471-2164-15-813, PMID: 25257245 PMC4180962

[ref26] Letunic I. Bork P. (2021). Interactive tree Of life (iTOL) v5: an online tool for phylogenetic tree display and annotation. Nucleic Acids Res. 49, W293–W296. doi: 10.1093/nar/gkab301, PMID: 33885785 PMC8265157

[ref27] Li H. (2012). Seqtk toolkit for processing sequences in FASTA/Q formats. GitHub 767:69.

[ref28] Li L. G. Huang Q. Yin X. Zhang T. (2020). Source tracking of antibiotic resistance genes in the environment - challenges, progress, and prospects. Water Res. 185:116127. doi: 10.1016/j.watres.2020.116127, PMID: 33086465

[ref29] Li D. Luo R. Liu C.-M. Leung C.-M. Ting H.-F. Sadakane K.. (2016). MEGAHIT v1.0: A fast and scalable metagenome assembler driven by advanced methodologies and community practices. Methods 102, 3–11. doi: 10.1016/j.ymeth.2016.02.020, PMID: 27012178

[ref30] Liu Y. X. Qin Y. Chen T. Lu M. Qian X. Guo X.. (2021). A practical guide to amplicon and metagenomic analysis of microbiome data. Protein Cell 12, 315–330. doi: 10.1007/s13238-020-00724-8, PMID: 32394199 PMC8106563

[ref800] Lu J. Breitwieser F. P. Thielen P. Salzberg S. L. (2017). Bracken: estimating species abundance in metagenomics data. Peerj Computer Science. 3:e104. doi: 10.7717/peerj-cs.104 PMC12016282 40271438

[ref500] Lu T. Wu R. B. Zhang Z. S. (2021). The Revalidation of Pardosa agraria Tanaka, 1985 (Lycosidae: Pardosa). Acta Arachnol. Sinica 30, 54–58. doi: 10.3969/j.issn.1005-9628.2021.01.009

[ref31] Moran N. A. Ochman H. Hammer T. J. (2019). Evolutionary and ecological consequences of gut microbial communities. Annu. Rev. Ecol. Evol. Syst. 50, 451–475. doi: 10.1146/annurev-ecolsys-110617-062453, PMID: 32733173 PMC7392196

[ref32] Oksanen J. Kindt R. Legendre P. O’Hara B. Stevens M. H. H. Oksanen M. J.. (2007). The vegan package. Comm. Eco. Package 10:719.

[ref33] Olm M. R. Brown C. T. Brooks B. Banfield J. F. (2017). dRep: a tool for fast and accurate genomic comparisons that enables improved genome recovery from metagenomes through de-replication. ISME J. 11, 2864–2868. doi: 10.1038/ismej.2017.126, PMID: 28742071 PMC5702732

[ref34] Parks D. H. Chuvochina M. Chaumeil P.-A. Rinke C. Mussig A. J. Hugenholtz P. (2020). A complete domain-to-species taxonomy for bacteria and archaea. Nat. Biotechnol. 38, 1079–1086. doi: 10.1038/s41587-020-0501-8 32341564

[ref35] Parks D. H. Tyson G. W. Hugenholtz P. Beiko R. G. (2014). STAMP: statistical analysis of taxonomic and functional profiles. Bioinformatics 30, 3123–3124. doi: 10.1093/bioinformatics/btu494, PMID: 25061070 PMC4609014

[ref36] Patro R. Duggal G. Love M. I. Irizarry R. A. Kingsford C. (2017). Salmon provides fast and bias-aware quantification of transcript expression. Nat. Methods 14, 417–419. doi: 10.1038/nmeth.4197 28263959 PMC5600148

[ref37] Plotka M. Kaczorowska A. K. Morzywolek A. Makowska J. Kozlowski L. P. Thorisdottir A.. (2015). Biochemical characterization and validation of a catalytic site of a highly thermostable Ts2631 Endolysin from the Thermus scotoductus phage vB_Tsc2631. PLoS One 10:e0137374. doi: 10.1371/journal.pone.0137374, PMID: 26375388 PMC4573324

[ref38] Quince C. Walker A. W. Simpson J. T. Loman N. J. Segata N. (2017). Shotgun metagenomics, from sampling to analysis. Nat. Biotechnol. 35, 833–844. doi: 10.1038/nbt.3935, PMID: 28898207

[ref43] R Core Team (2013). R: A language and environment for statistical computing. Vienna: R Foundation for Statistical Computing. Available at: https://scholar.google.com/scholar_lookup?title=A+Language+and+Environment+for+Statistical+Computing%2E&publication_year=2012

[ref39] Ruiz C. McCarley A. Espejo M. L. Cooper K. K. Harmon D. E. (2019). Comparative genomics reveals a well-conserved intrinsic Resistome in the emerging multidrug-resistant pathogen *Cupriavidus gilardii*. mSphere 4:e00631-19. doi: 10.1128/mSphere.00631-19 PMC6796972 31578249

[ref40] Sakon H. Nagai F. Morotomi M. Tanaka R. (2008). Sutterella parvirubra sp. nov. and *Megamonas funiformis* sp. nov., isolated from human faeces. Int. J. Syst. Evol. Microbiol. 58, 970–975. doi: 10.1099/ijs.0.65456-0, PMID: 18398204

[ref41] Shafi S. Kamili A. N. Shah M. A. Bandh S. A. Dar R. (2017). Dynamics of bacterial class bacilli in the deepest valley lake of Kashmir-the Manasbal Lake. Microb. Pathog. 104, 78–83. doi: 10.1016/j.micpath.2017.01.018, PMID: 28087491

[ref42] Sheffer M. M. Uhl G. Prost S. Lueders T. Urich T. Bengtsson M. M. (2020). Tissue- and population-level microbiome analysis of the wasp spider Argiope bruennichi identified a novel dominant bacterial symbiont. Microorganisms 8:8. doi: 10.3390/microorganisms8010008, PMID: 31861544 PMC7023434

[ref44] Thombre R. S. Shivakarthik E. Sivaraman B. Vaishampayan P. A. Seuylemezian A. Meka J. K.. (2019). Survival of Extremotolerant bacteria from the Mukundpura meteorite impact crater. Astrobiology 19, 785–796. doi: 10.1089/ast.2018.1928, PMID: 31081685

[ref46] Uritskiy G. V. DiRuggiero J. Taylor J. (2018). MetaWRAP-a flexible pipeline for genome-resolved metagenomic data analysis. Microbiome 6:158. doi: 10.1186/s40168-018-0541-1, PMID: 30219103 PMC6138922

[ref47] Villanueva R. A. M. Chen Z. J. (2019). ggplot2: elegant graphics for data analysis. Measure. Interdis. Res. Perspectives 17, 160–167. doi: 10.1080/15366367.2019.1565254

[ref48] Wilpiszeski R. L. Zhang Z. House C. H. (2019). Biogeography of thermophiles and predominance of Thermus scotoductus in domestic water heaters. Extremophiles 23, 119–132. doi: 10.1007/s00792-018-1066-z, PMID: 30536130

[ref49] Wood D. E. Lu J. Langmead B. (2019). Improved metagenomic analysis with kraken 2. Genome Biol. 20. doi: 10.1186/s13059-019-1891-0, PMID: 31779668 PMC6883579

[ref50] World Spider Catalog (2021). Version 22.5. Natural History Museum Bern. Available at https://wsc.nmbe.ch/, (Accessed June 25, 2021).

[ref51] Wu T. Hu E. Xu S. Chen M. Guo P. Dai Z.. (2021). Clusterprofiler 4.0: A universal enrichment tool for interpreting omics data. Innovations 2:100141. doi: 10.1016/j.xinn.2021.100141 PMC8454663 34557778

[ref52] Wu Y.-W. Simmons B. A. Singer S. W. (2016). MaxBin 2.0: an automated binning algorithm to recover genomes from multiple metagenomic datasets. Bioinformatics 32, 605–607. doi: 10.1093/bioinformatics/btv638, PMID: 26515820

[ref53] Zhang J. Liu Y.-X. Zhang N. Hu B. Jin T. Xu H.. (2019). NRT1.1B is associated with root microbiota composition and nitrogen use in field-grown rice. Nat. Biotechnol. 37:676. doi: 10.1038/s41587-019-0104-4, PMID: 31036930

[ref54] Zhang H. Yohe T. Huang L. Entwistle S. Wu P. Yang Z.. (2018a). dbCAN2: a meta server for automated carbohydrate-active enzyme annotation. Nucleic Acids Res. 46, W95–W101. doi: 10.1093/nar/gky418 29771380 PMC6031026

[ref55] Zhang L. Yun Y. Hu G. Peng Y. (2018b). Insights into the bacterial symbiont diversity in spiders. Ecol. Evol. 8, 4899–4906. doi: 10.1002/ece3.4051 29876068 PMC5980269

