# Corrigendum: Diversity and Function of Wolf Spider Gut Microbiota Revealed by Shotgun Metagenomics

**DOI:** 10.3389/fmicb.2022.849170

**Published:** 2022-02-02

**Authors:** Runbiao Wu, Luyu Wang, Jianping Xie, Zhisheng Zhang

**Affiliations:** ^1^Key Laboratory of Eco-environments in Three Gorges Reservoir Region (Ministry of Education), School of Life Sciences, Southwest University, Chongqing, China; ^2^Key Laboratory of Freshwater Fish Reproduction and Development (Ministry of Education), School of Life Sciences, Southwest University, Chongqing, China

**Keywords:** shotgun metagenomic sequencing, spiders, host-bacterial interaction, symbiosis, microbiome

In the original article ***Abstract***, there was the following error: ***Approximately 27.3% of the gut microbiota of P. agraria comprises Proteobacteria, and approximately 34.5% of the gut microbiota of P. laura comprises Firmicutes***.

The following correction has been applied:

Approximately 27.3% of the gut microbiota of *P. agraria* comprises Proteobacteria, and approximately 34.4% of the gut microbiota of *P. laura* comprises Firmicutes.

In the original article Figure 4 **legend**, contained the following errors: **Ras guanyl-nuxleotide exchange factor activity** should be changed to **“Ras guanyl-nucleotide exchange factor activity,”** and **intracellular receptor signaling patway** should be changed to **“intracellular receptor signaling pathway.”**

**Figure 4:**
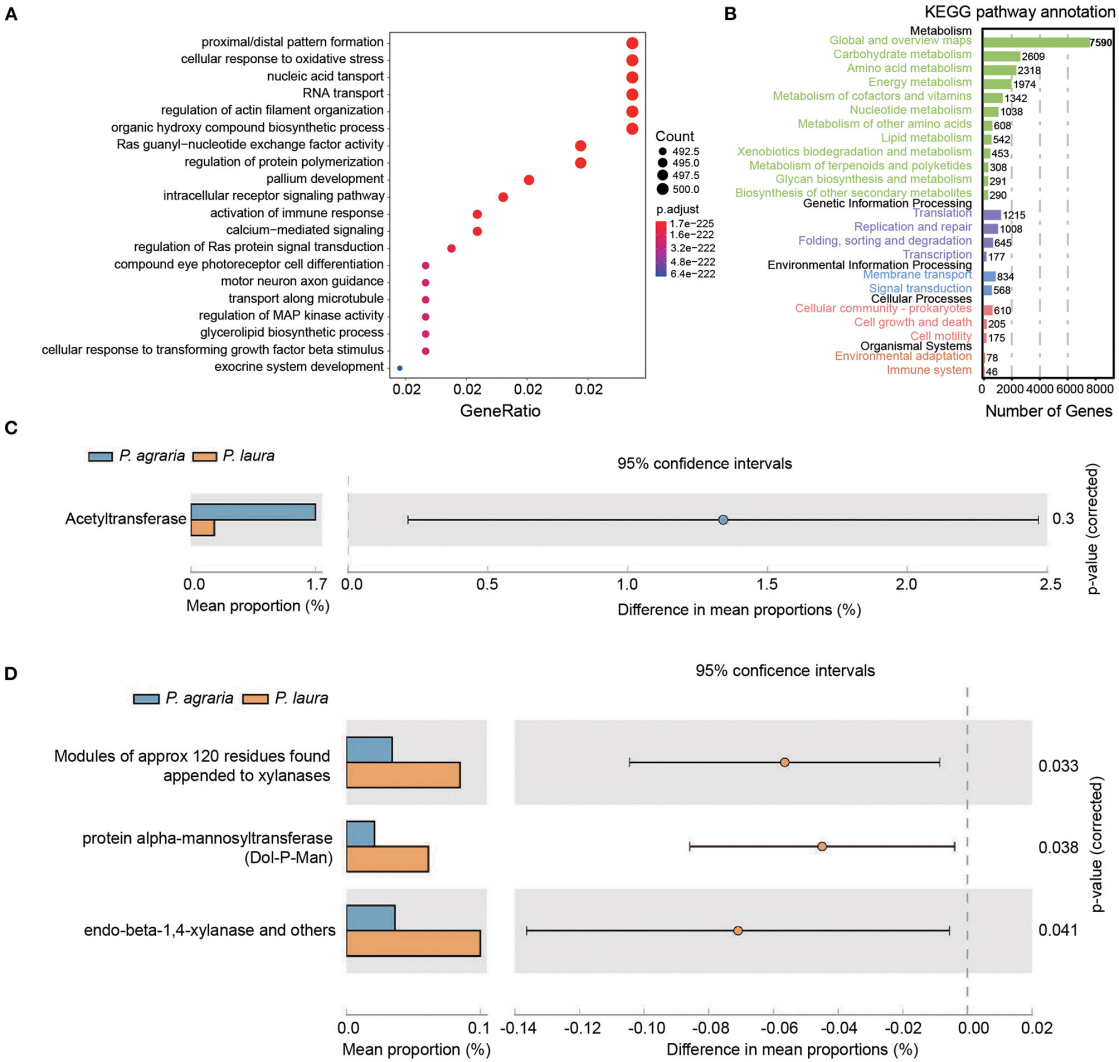
Functional annotation of the genes in gut bacteria of the two Pardosa species. **(A)** Genes that mapped to the COG category were annotated using eggnog. Top 20 of the categorized functional annotation enrichment predicted in the bubble chart. The size of the circle indicates the number of genes and the color represents the significance. **(B)** Kyoto Encyclopedia of Genes and Genomes functional category analysis was done using GhostKOALA. Rows indicate the number of genes annotated to the corresponding B-level pathway, columns in black font are the name of the classification. **(C)** Comparison of antibiotic resistance-associated genes in the two Pardosa species based on the resfam database. **(D)** Comparison of carbohydrate metabolism-associated genes in the two Pardosa species based on the carbohydrate-active enzyme database (dbCAN2).

In the original article, ***MATERIALS AND METHODS***, ***Assembly-Free Metagenomic Profiling, Metagenome Assembly, Gene Prediction, and Annotation***: there was the following error: ***Quality control of metagenomic sequencing data was performed using MultiQC v1.5 (Ewels et al., 2016) with parameter -t 20***.

The following correction has been applied: Quality control of metagenomic sequencing data was performed using FastQC v0.11.8^1^ with parameter -t 20 and MultiQC v1.10.1 (Ewels et al., 2016) with parameter -d.

^1^ http://www.bioinformatics.babraham.ac.uk/projects/fastqc/

In the original article ***RESULTS***, ***Identification of Microbiota From the Reads of Spider Metagenomes***, there were the following errors: ***The most abundant gut microbiota in P. agraria were bacteria, accounting for 99.68%, followed by archaea and viruses accounting for 0.3 and 0.02%, respectively (Figure 2A)***. ***In the gut of P***. ***laura, bacteria, archaea, and viruses accounted for 97.4, 2, and 0.6%, respectively (Figure 2B)***.

***In both species, bacteria from the phylum Proteobacteria were most abundant***, ***accounting for approximately 27.3% (127,304/466,552) in P. agraria and 34.5% (186,388/542,484) in P. laura (Figure 2C)***.

The following correction has been applied:

The most abundant gut microbiota in *P. agraria* were bacteria, accounting for 99.64%, followed by archaea and viruses accounting for 0.34 and 0.02%, respectively (Figure 2A). In the gut of *P. laura*, bacteria, archaea, and viruses accounted for 97.56, 1.8, and 0.64%, respectively (Figure 2B).

In both species, bacteria from the phylum Proteobacteria were most abundant, accounting for approximately 27.3% (127,304/466,552) in *P. agraria* and 34.4% (186,388/542,484) in *P. laura* (Figure 2C).

The authors apologize for the errors and state that this does not change the scientific conclusions of the article in any way. The original article has been updated.

## Publisher's Note

All claims expressed in this article are solely those of the authors and do not necessarily represent those of their affiliated organizations, or those of the publisher, the editors and the reviewers. Any product that may be evaluated in this article, or claim that may be made by its manufacturer, is not guaranteed or endorsed by the publisher.

